# Immunotherapy of Malignant Glioma by Noninvasive Administration of TLR9 Agonist CpG Nano‐Immunoadjuvant

**DOI:** 10.1002/advs.202103689

**Published:** 2022-03-07

**Authors:** Jingjing Wei, Di Wu, Songsong Zhao, Yu Shao, Yifeng Xia, Dawei Ni, Xinyun Qiu, Jinping Zhang, Jian Chen, Fenghua Meng, Zhiyuan Zhong

**Affiliations:** ^1^ Biomedical Polymers Laboratory College of Chemistry, Chemical Engineering and Materials Science College of Pharmaceutical Sciences and State Key Laboratory of Radiation Medicine and Protection Soochow University Suzhou 215123 P. R. China; ^2^ Institute of Functional Nano & Soft Materials (FUNSOM) Soochow University Suzhou 215123 P. R. China; ^3^ Institutes of Biology and Medical Sciences (IBMS) Soochow University Suzhou 215123 P. R. China; ^4^ Chinese Institute for Brain Research, Beijing Research Unit of Medical Neurobiology Chinese Academy of Medical Sciences (No. 2019RU003) Beijing 102206 P. R. China

**Keywords:** brain tumor, immunoadjuvant, immunotherapy, polymersomes, targeted delivery

## Abstract

Immunotherapy with toll like receptor 9 (TLR9) agonist CpG ODN offers an emergent strategy to treat life‐threatening malignant glioma. CpG is typically applied invasively by intracranial and intrathecal administration which induces not only poor compliance and lessened potency but also possibly strong adverse effects and immunotoxicity. Here, it is reported that immunotherapy of murine LCPN glioma is greatly boosted by polymersome‐steered intravenous and intranasal brain delivery of CpG. CpG is efficiently loaded in apolipoprotein E peptide‐directed polymersomes to give blood‐brain barrier permeable and glioma and cervical lymph node‐homing CpG nano‐immunoadjuvant (t‐NanoCpG) which strongly stimulates the maturation of dendritic cells, antigen cross‐presentation, and production of proinflammatory cytokines in vivo. Intriguingly, both intravenous and intranasal administration of t‐NanoCpG brings about significant survival benefits in murine LCPN glioma‐bearing mice while free CpG and nontargeted CpG nano‐immunoadjuvant (NanoCpG) afford modest therapeutic effects. Moreover, combination of t‐NanoCpG with radiotherapy further boosts the immunotherapeutic effects leading to more improved survival rate of mice. This intelligent brain‐permeable nano‐immunoadjuvant provides a new, minimally invasive and highly potent strategy for immunotherapy of glioma.

## Graphical abstract

This work discloses a blood‐brain‐barrier (BBB)‐permeable, glioma, and cervical lymph node‐homing CpG nano‐immunoadjuvant (t‐NanoCpG) that either alone or combining with radiotherapy induces strong immunotherapeutic effects, leading to significant survival benefits in malignant murine glioma‐bearing mice. t‐NanoCpG provides a new, minimally invasive, and highly potent strategy for immunotherapy of glioma.

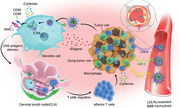

## 1 Introduction

Malignant glioma with a highly invasive nature has poor prognosis with a five‐year survival rate of less than 5% worldwide.^[^ ^1^ ^]^ Tumor immunotherapy by activating host's immune response to repress tumor and improve survival has brought revolutionary breakthroughs for cancers including melanoma and non‐small cell lung cancer.^[^ ^2^ ^]^ However, there has been little progress in the immunotherapy of glioblastoma, as evidenced by the failure of recent phase III clinical trials with anti‐PD‐1 therapy.^[^ ^3^ ^]^ On the one hand, the existence of blood‐brain barrier (BBB) sets a blockade for therapeutic drugs including immunotherapeutic agents from reaching glioma sites.^[^ ^4^ ^]^ On the other hand, malignant glioma is an immunologically “cold” tumor which is infiltrated with a large number of immune‐suppressive cells such as tumor‐associated macrophages (TAM) and myeloid‐derived suppressor cells (MDSC) in the highly innate and adaptive immune‐resistant tumor microenvironment (TME), leading to minimal immune response.^[^ ^5^ ^]^ However, therapeutic vaccines against either the IDH1R132H mutation or tailored neoantigens have recently shown encouraging immune responses and antitumor activity in clinical trials,^[^ ^6^ ^]^ suggesting that robust immune response can be elicited if proper immunogens and immune adjuvants were used.

CpG ODN, an agonist of the toll‐like receptor 9 (TLR9) that can potentiate immunogenicity, has been widely used as immune adjuvant in cancer immunotherapy field in both clinical trials and preclinical studies.^[^ ^7^ ^]^ For glioma treatment, CpG is invasively applied by intracranial (i.c.) or intrathecal (i.t.) administration, which induces not only poor compliance and lessened potency owing to inefficient cellular uptake and fast degradation in vivo but also possibly strong adverse effects and immunotoxicity.^[^ ^8^ ^]^ Besides, to avoid swelling and edema, glioma patients are often given large amounts of immunosuppressant dexamethasone after tumor resection, which has been shown to substantially reduce T‐cell infiltration and response to tumor neoantigen vaccines.^[^ ^9^ ^]^ This has led to severe limitations in the delivery of immune adjuvants such as CpG by surgical means. The postsurgery local administration of immunoadjuvants in hydrogels, either as a monotherapy or combination therapy, could be a better though invasive strategy for glioma therapy.^[^ ^10^ ^]^ Nanoparticles that are able to protect immunoadjuvant in circulation and enhance its cell uptake have shown to boost adjuvant‐based immunotherapy for melanoma and colorectal cancer.^[^ ^11^ ^]^ Little progress has, however, been made on minimally invasive adjuvant‐based immunotherapy for malignant glioma.

**Figure 1:**
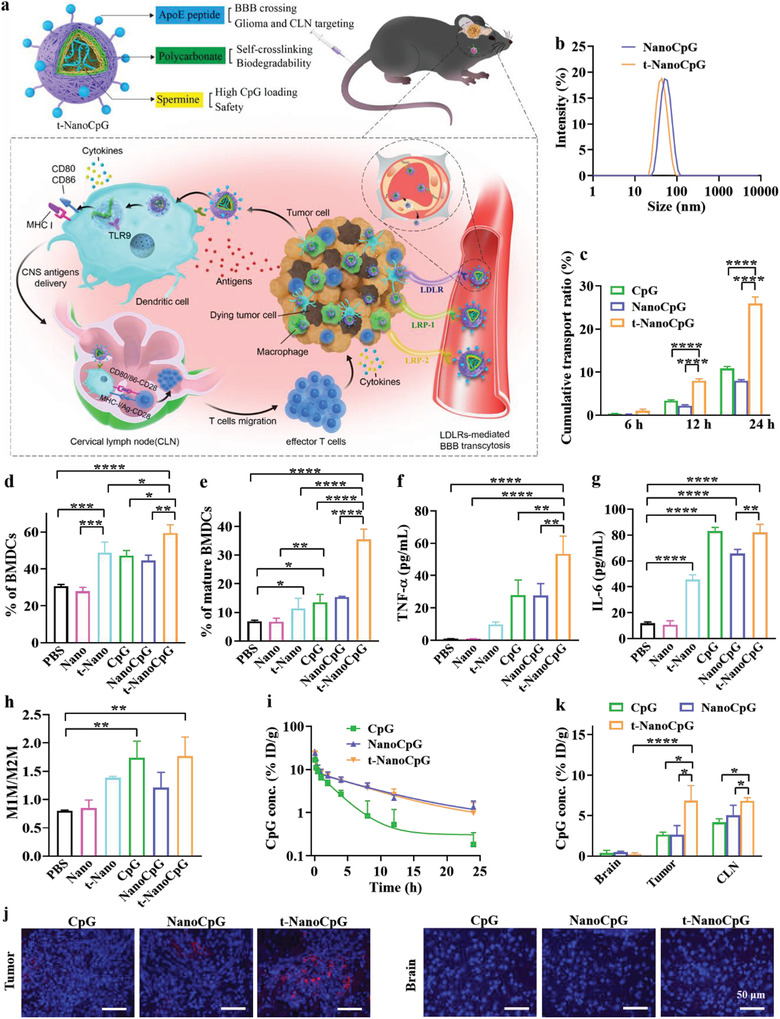
Design and characterization of apolipoprotein E peptide‐functionalized polymersome CpG nanoformulation (t‐NanoCpG) for intravenous brain delivery of CpG. a) Schematic of t‐NanoCpG structure and strategy for enhancing intracellular delivery of CpG in orthotopic glioma and cervical lymph nodes. b) Size distributions of t‐NanoCpG and NanoCpG (nontargeted control). c) In vitro BBB transcytosis assay of CpG, NanoCpG and t‐NanoCpG using bEnd.3 murine endothelial cell monolayer as a BBB model (*n* = 3). Quantitative determination of the percentage of d) CD11c^+^ BMDCs and mature e) BMDCs (CD80^+^CD86^+^) out of total BMDCs by flow cytometry (*n* = 3). Concentrations of secreted proinflammatory f) TNF‐*α* and g) IL‐6 by BMDCs determined using ELISA kits (*n* = 3). h) Quantitative determination of the ratio of M1 phenotype macrophages to M2 phenotype macrophages (M1M/M2M) (*n* = 3). i) In vivo pharmacokinetics of CpG formulations (*n* = 3). j) The distribution of CpG formulations in tumor and brain tissues. k) Quantitative analyses of in vivo bio‐distribution of CpG in brain, tumor, and CLN (*n* = 3). For (c), (i), (j), (k), CpG‐Cy3 was used for facile quantification. *p < 0.05, **p < 0.01, ***p < 0.001, ****p < 0.0001.

Here, we show that intravenous (i.v.) or intranasal (i.n.) administration of apolipoprotein E peptide (ApoE)‐directed polymersome CpG nanoformulation (t‐NanoCpG) mediates efficient brain delivery of CpG, inducing strong immunotherapy of highly malignant murine LCPN glioma (**Figure** **1**a). t‐NanoCpG is based on a disulfide‐crosslinked chimeric biodegradable polycarbonate vesicle, which has shown to achieve robust loading and intracellular redox‐triggered release of small interfering RNA,^[^ ^12^ ^]^ and a short ApoE peptide (LRKLRKRLLLRKLRKRLLC), which has shown to promote brain delivery.^[^ ^13^ ^]^ Intriguingly, t‐NanoCpG not only exhibits blood‐brain‐barrier (BBB)‐permeability but also homes to glioma and cervical lymph node (CLN), strongly stimulating the maturation of dendritic cells (DCs), antigen cross‐presentation and production of proinflammatory cytokines in vivo and bringing about significant survival benefits for LCPN glioma‐bearing mice. The combination of t‐NanoCpG with radiotherapy further boosts immunotherapy for LCPN glioma. This intelligent brain‐permeable nano‐immunoadjuvant provides a new, minimally invasive and highly potent strategy for immunotherapy of glioma.

## 2 Results and Discussion

### 2.1 Preparation and In Vitro Characterization of t‐NanoCpG

t‐NanoCpG is co‐self‐assembled from poly(ethylene glycol)‐polycarbonate‐spermine (Figure S1, Supporting Information), ApoE‐poly(ethylene glycol)‐polycarbonate (Figure S2, Supporting Information), and CpG in aqueous media. Notably, t‐NanoCpG showed a size of 44 nm with negative surface charge of −5.2 mV and quantitative loading of CpG at a feeding content of 10 wt% (Figure 1b and Table S1, Supporting Information), in line with the active loading of CpG into the vesicular interior via electrostatic and hydrogen bonding interactions with positively charged spermine. A high loading efficiency of 93.2% was obtained even at a CpG feeding content of 20 wt%. CpG loading had little influence on the physiochemical properties of t‐NanoCpG. NanoCpG (nontargeted control) exhibited similar CpG loading and size profile. t‐NanoCpG exhibited good colloidal stability against dilution, 10% FBS, and 7‐day storage (Figure S3, Supporting Information). The in vitro release studies showed accelerated release of CpG from t‐NanoCpG at pH 4.0 than at pH 6.5 and 7.4.

To effectively treat glioma, penetrating BBB is one of the prerequisites. The BBB transcytosis behavior of t‐NanoCpG was investigated in bEnd.3 murine endothelial cell monolayer model using Cy3‐labeled CpG (CpG‐Cy3) as a probe. The results showed significantly enhanced transcytosis of t‐NanoCpG compared with NanoCpG and free CpG at 12 and 24 h (Figure 1c), supporting that ApoE peptide mediates efficient BBB penetration of t‐NanoCpG likely via binding to LDLRs.^[^ ^13^, ^14^ ^]^ As CpG plays its immunotherapeutic role mainly by activating antigen presentation cells (APCs), we next evaluated the effect of t‐NanoCpG on the maturation of bone marrow‐derived dendritic cells (BMDCs) in vitro. Interestingly, t‐NanoCpG group revealed 59% BMDCs (CD11c^+^) out of all measured cells, which was significantly higher than NanoCpG and free CpG groups (Figure 1d and Figure S4, Supporting Information). The percentage of mature BMDCs (CD80^+^CD86^+^) out of all measured cells in t‐NanoCpG group (35%) was 2.3–5‐fold higher than control groups (Figure 1e and Figure S4, Supporting Information). Accordingly, t‐NanoCpG stimulated significant secretion of proinflammatory tumor necrosis factor alpha (TNF‐*α*) (Figure 1f) and interleukin‐6 (IL‐6) (Figure 1g). The treatment of bone marrow‐derived macrophages (BMDMs) with t‐NanoCpG led to an increased M1M/M2M which was comparable to that with free CpG but higher than NanoCpG and all other controls (Figure 1h). Notably, empty polymersomes, t‐Nano, were shown to induce certain immune responses but significantly less compared with t‐NanoCpG. The above results indicate that t‐NanoCpG is able to enhance BBB penetration as well as APC maturation.

### 2.2 Pharmacokinetics and Brain Distribution of t‐NanoCpG in Orthotopic Murine LCPN Glioma Model

We next studied the pharmacokinetics and brain distribution of t‐NanoCpG in orthotopic murine LCPN glioma‐bearing mice following i.v. injection. Notably, both NanoCpG and t‐NanoCpG exhibited a prolonged elimination half‐life (t_1/2, *β*_) of about 7 h (Figure 1i). To build orthotopic murine glioma model, murine LCPN cells were injected intracranially to female C57BL/6 mice using stereotactic technique. LCPN cells were derived from a P5 male mouse neural stem cell with deletions of two most frequently mutated genes in human gliomas, P53 and NF1, by CRISPR/Cas9 technology. The cells elicit strong immunogenicity if transplanted into female mice because of the existence of the male specific H‐Y antigen. The immunohistochemical staining of LCPN tumor slices with anti‐GFAP (a representative marker of glial cells) and anti‐Ki67 (a representative marker of proliferating cells) antibodies showed GFAP^+^ and Ki67^+^ feature, confirming its glioma nature (Supporting Information). Intriguingly, C57BL/6 mice bearing orthotopic LCPN glioma treated with t‐NanoCpG displayed pronounced Cy3 fluorescence in the tumor tissues but not in normal brain tissues (Figure 1j). The quantitative analyses revealed that t‐NanoCpG achieved a remarkable accumulation of about 6.8% ID/g in both glioma and cervical lymph node (CLN), which was significantly higher than NanoCpG and free CpG groups, and than that in normal brain tissues (Figure 1k, ****p), pointing to a high glioma and CLN‐selectivity of t‐NanoCpG. The biodistribution results demonstrated that t‐NanoCpG had significantly lower accumulation in the kidney while similar or slightly reduced accumulation in the liver, heart, spleen and lung compared with NanoCpG and free CpG (Figure S5, Supporting Information). The enhanced accumulation of t‐NanoCpG in LCPN glioma was attributable to its LDLRs‐mediated BBB penetration and high affinity to LCPN glioma cells. Besides, t‐NanoCpG with a size of 44 nm accumulated preferably in CLN, in accordance to the report that nanoparticles of < 50 nm were capable of selectively accumulating inside lymph nodes.^[^ ^15^ ^]^ It is known that CLN is crucial to amplify immune response of glioma immunotherapy. Central nervous system (CNS) antigens are sensed by draining cerebrospinal fluid (CSF) and delivered by meningeal lymphatics to CLN.^[^ ^16^ ^]^ Glioma and CLN‐homing properties of t‐NanoCpG via i.v. injection provide more opportunities for boosting immune response in glioma.

### 2.3 Immunotherapeutic Efficacy of t‐NanoCpG in LCPN Glioma Model via i.v. Injection

**Figure 2:**
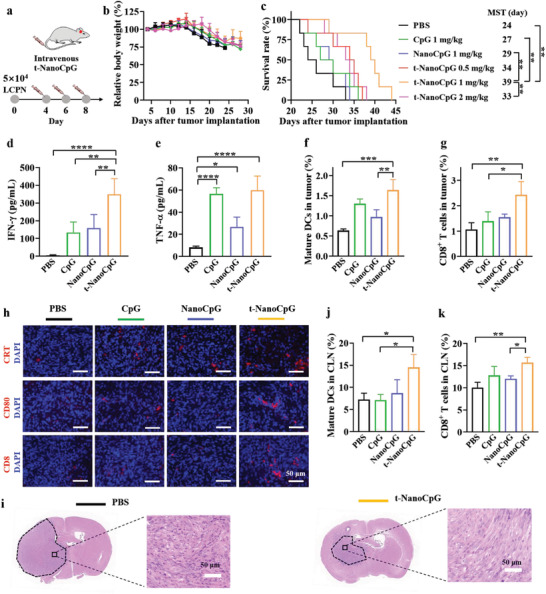
Intravenous injection of t‐NanoCpG enhances immunotherapeutic efficacy in orthotopic LCPN model. a) Intravenous administration scheme. LCPN‐bearing mice were i.v. injected with CpG (1 mg kg^−1^), NanoCpG (1 mg kg^−1^), or t‐NanoCpG (0.5, 1, or 2 mg kg^−1^) on day 4, 6, and 8 after tumor implantation. b) Relative body weight (*n* = 6). c) Survival curves (*n* = 6). The plasma concentrations of d) IFN‐*γ* and e) TNF‐*α* of LCPN‐bearing mice following different treatments (at a fixed dosage of 1 mg kg^−1^) on day 9 after tumor implantation (*n* = 4). Analysis of immune cells in tumors of orthotopic LCPN‐bearing mice treated with different formulations (*n* = 3). Percentages of f) CD8^+^ T cells and mature g) DCs (CD11c^+^CD86^+^CD80^+^) in tumors. h) Immunohistochemical staining using CRT, CD80, and CD8 antibody of brain tumor sections on day 9 postimplantation. i) Hematoxylin‐eosin (H&E) staining of brain tumor sections on day 23 postimplantation. Analysis of immune cells in cervical lymph nodes (CLN) of orthotopic LCPN‐bearing mice treated with different formulations. Percentages of j) CD8^+^ T cells and k) mature DCs (CD11c^+^CD86^+^CD80^+^) in CLN (*n* = 3). *p < 0.05, **p < 0.01, ***p < 0.001, ****p < 0.0001.

Inspired by its glioma and CLN‐selectivity and strong BMDC activation, we investigated the immunotherapeutic efficacy of t‐NanoCpG against an orthotopic murine LCPN glioma model. Although this model elicited strong immunogenicity in female mice, it is not sensitive to the immune checkpoint blockade therapy (anti‐PD‐1 and anti‐CTLA‐4 antibodies) (Figure S6, Supporting Information), which is consistent to the failure of recent phase III clinical trials in human.^[^ ^3^ ^]^ It was reported that malignant glioma‐infiltrating CD8^+^ T cells are associated with patient outcomes while usually expressed the suppressive marker IDO‐1 and TIM‐3 rather than PD‐1.^[^ ^17^ ^]^ Luciferase‐transfected LCPN cells (LCPN‐Luc) were used to build orthotopic LCPN‐Luc model to visualize the progression of glioma using in vivo imaging. The results revealed fast tumor invasion and a clear correlation between tumor growth and body weight loss (Figure S7, Supporting Information). The orthotopic LCPN glioma mice were i.v. injected with t‐NanoCpG at varying dosages of 0.5, 1 or 2 mg kg^−1^ on day 4, 6, and 8 post‐tumor implantation (*n* = 6) (**Figure** **2**a). Free CpG and NanoCpG at 1 mg kg^−1^ were used as controls. The orthotopic LCPN glioma is highly malignant and the mice without treatment quickly lost weight as from day 12 post‐tumor implantation (Figure 2b). The i.v. administered t‐NanoCpG greatly retarded the reduction in body weight of the mice (Figure 2b), indicating its good safety as well as effective inhibition of glioma invasion. Remarkably, the survival curves showed that t‐NanoCpG at 1 mg kg^−1^ significantly increased the median survival time (MST) to 39 d (** *p*). While free CpG and NanoCpG at the same dosage caused modest improvement of MST (27 and 29 d, respectively) compared with PBS (24 d) (Figure 2c), signifying the crucial role of BBB‐crossing and glioma/CLN‐homing of t‐NanoCpG in glioma immunotherapy. It is interesting to note that t‐NanoCpG exhibited the best survival benefit at 1 mg kg^−1^ and a higher dose of t‐NanoCpG (2 mg kg^−1^) compromised the therapeutic effects, likely as a result of over‐stimulation.^[^ ^18^ ^]^ The empty polymersomes, t‐NanoCpG, had little effect on mice survival rate (Figure S8, Supporting Information).

To elucidate the role of t‐NanoCpG in the induction of immune‐mediated anti‐glioma activity in vivo, we analyzed the cytokines in the plasma and immune TME. TNF‐*α* and interferon gamma (IFN‐*γ*) in plasma at 24 h post‐treatment (day 9) were measured. The results showed that t‐NanoCpG group had 70 and sevenfold elevation of IFN‐*γ* and TNF‐*α*, respectively, compared with PBS group (Figure 2d,e). In comparison, NanoCpG group had significantly lower plasma IFN‐*γ* and TNF‐*α* level. Free CpG group while having a comparable TNF‐*α* to t‐NanoCpG group exhibited a markedly lower IFN‐*γ*. These results confirm that i.v. injection of t‐NanoCpG can stimulate proper immune response in orthotopic murine LCPN glioma model.

To confirm the stimulatory effect of t‐NanoCpG on immune TME, we quantified the tumor infiltrating immune cells on day 9 using flow cytometry. When recognizing exogenous CpG or the danger associated molecule patterns, e.g., CRT and HMGB1 released by the necrotic or apoptotic tumor cells, DCs can be activated and express elevated levels of molecules involved in antigen presentation such as MHC and co‐stimulatory molecules, CD80 and CD86. Notably, t‐NanoCpG treated mice showed a considerable increase of mature CD80^+^CD86^+^ DCs (Figure 2f and Figure S9a, Supporting Information), and significantly more CD8^+^ T cells than free CpG and NanoCpG group (Figure 2g and Figure S9b, Supporting Information) in the TME. Moreover, we performed immunofluorescence staining of glioma‐containing brain slices harvested on day 9 post‐tumor implantation. Figure 2h shows obviously large‐scale anti‐CD80 and anti‐CD8‐positive region in t‐NanoCpG group, which correspond to enrichment of activated APCs and CD8^+^ T cells, respectively. Furthermore, the immunofluorescence staining showed the presence of more CRT in the tumor of t‐NanoCpG group than free CpG and NanoCpG groups, pointing to the immunogenic cell death (ICD) of tumor cells. The ICD could be enhanced by CpG or a result of overgrowth of tumor cells.^[^ ^19^ ^]^ In addition, the hematoxylin‐eosin (H&E) staining of whole brain slices harvested on day 23 illustrated that t‐NanoCpG group had much smaller tumor size and less dense tumor texture than PBS control (Figure 2i).

CLN is important immune organ for orthotopic glioma therapy. Of note, t‐NanoCpG also induced significant increase of mature DCs and CD8^+^ T cells in the CLN compared with free CpG and NanoCpG (Figure 2j,k and Figure S9c,d, Supporting Information), confirming its great immune‐activation capacity. It is known that the antigen cross‐presentation by DCs is essential for the initiation of CD8^+^ T cell responses.^[^ ^20^ ^]^ DCs could efficiently cross‐present antigens after TLR9 activation by CpG at late endosomes.^[^ ^21^ ^]^ To activate TLR9, CpG must be taken up by DCs that over express LRP‐1.^[^ ^22^ ^]^ On the other hand, studies have shown that the specialized cross presenting function of CD8*α* ^+^ DCs was due to their ability to endocytose dying cells,^[^ ^23^ ^]^ and DCs did not cross present antigens from dead cells unless stimulated by CpG before antigen capture.^[^ ^24^ ^]^ Here, our results suggest that t‐NanoCpG having the disulfide‐crosslinked polymersome membrane as compared to free CpG would facilitate more entry of CpG to DCs for better TLR9 activation and antigen cross‐presentation for CD8^+^ T cell responses, thus trigger innate and adaptive immune responses within the TME and CLN and play antitumor roles in a synchronized manner. The blood biochemistry and blood routine analysis disclosed that intravenous injection of t‐NanoCpG and NanoCpG induced little damage to main organs (Figure S10, Supporting Information). There was no statistical difference in all parameters between t‐NanoCpG group and healthy control group.

We further explored the immunotherapeutic efficacy of t‐NanoCpG in orthotopic GL261‐luc glioma‐bearing mice. The results demonstrated that t‐NanoCpG induced the best tumor inhibition (Figure S11, Supporting Information). The TME immune analyses revealed that t‐NanoCpG significantly elevated the proportions of CD8^+^ T cells, CD4^+^ T cells, and DCs (CD11c^+^) in the glioma. These results confirm that t‐NanoCpG elicits also enhanced immunotherapeutic efficacy in syngeneic model of glioma.

### 2.4 Immunotherapeutic Efficacy of t‐NanoCpG in LCPN Glioma Model via Intranasal Injection

**Figure 3:**
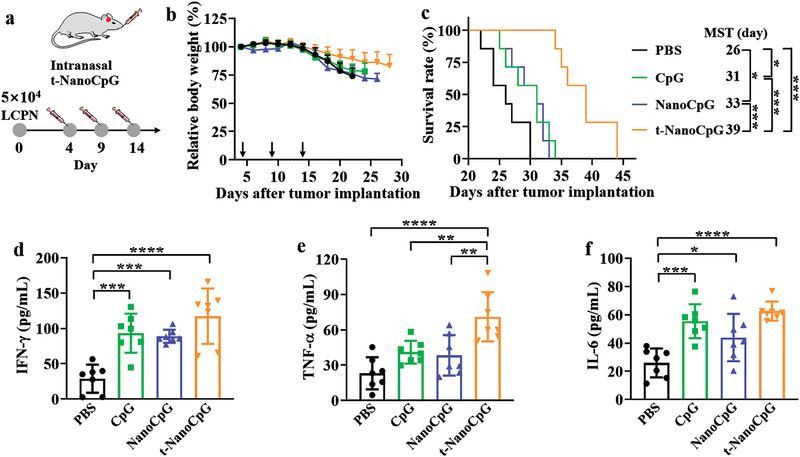
Intranasal injection of t‐NanoCpG enhances immunotherapeutic efficacy in orthotopic LCPN model. a) Intranasal administration scheme. The LCPN‐bearing mice were i.n. injected with CpG, NanoCpG, or t‐NanoCpG (0.5 mg kg^−1^) at 4, 9, and 14 days after tumor implantation. b) Relative body weight (*n* = 7). c) Survival curves (*n* = 7). Plasma concentration of d) IFN‐*γ*, e) TNF‐*α*, and f) IL‐6 of LCPN‐bearing mice following different treatments on day 15 post‐tumor implantation (*n* = 7). *p < 0.05, **p < 0.01, ***p < 0.001, ****p < 0.0001.

In addition to i.v. administration, intranasal (i.n.) injection is another minimally invasive approach that has been employed to bypass BBB by olfactory bulb pathway^[^ ^25^ ^]^ and treat brain‐associated diseases such as Alzheimer's disease^[^ ^26^ ^]^ and Parkinson's disease.^[^ ^27^ ^]^ Here, we also investigated the therapeutic effect of t‐NanoCpG by i.n. injection. The orthotopic LCPN mice were i.n. injected with t‐NanoCpG, NanoCpG, or CpG at a dosage of 0.5 mg kg^−1^ on day 4, 9, and 14 post‐tumor implantation (*n* = 7) (**Figure** **3**a). We employed a lower dose and dosing frequency for i.n. injection than i.v. administration. Figure 3b clearly shows lessened body weight loss for t‐NanoCpG group, certifying its effectiveness in retarding glioma invasion. Accordingly, t‐NanoCpG group showed a markedly prolonged MST of 39 d while free CpG and NanoCpG groups exhibited limited improvement of MST (31 and 33 d, respectively) compared with PBS control (26 d) (Figure 3c). The i.n. injection of t‐NanoCpG was shown to greatly stimulate the production of plasma proinflammatory cytokines like IFN‐*γ*, TNF‐*α*, and IL‐6 (Figure 3d–f). Notably, t‐NanoCpG, induced significantly more elevated level of TNF‐*α* than both free CpG and NanoCpG, corroborating that t‐NanoCpG is a superior immunoadjuvant for glioma immunotherapy.

### 2.5 Combination Therapy of t‐NanoCpG with Radiotherapy in Orthotopic LCPN Glioma Model

**Figure 4:**
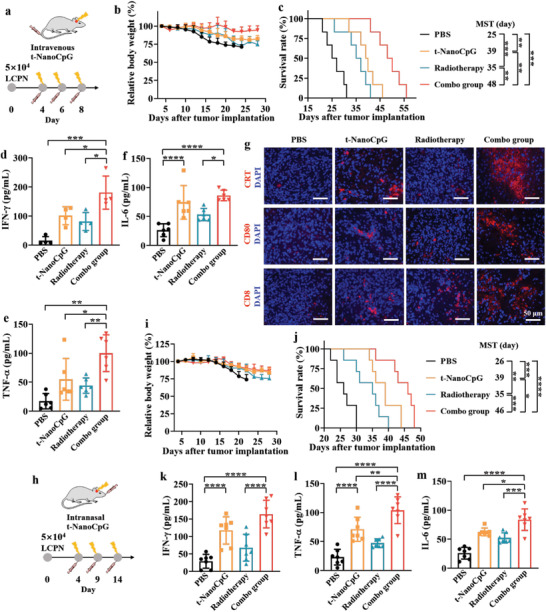
The combination of intravenous or intranasal injection of t‐NanoCpG with radiotherapy further enhances the immunotherapeutic efficacy of orthotopic LCPN glioma. a) The i.v. combo treatment scheme. On day 4, 6, and 8 after tumor implantation, LCPN‐bearing mice were subject to 3 Gy radiotherapy and 6 h later i.v. injection with t‐NanoCpG (1 mg kg^−1^). b) Relative body weight (*n* = 6). c) Survival curves (*n* = 6). The plasma concentration of d) IFN‐*γ*, e) TNF‐*α*, and f) IL‐6 on day 9 in i.v. combo therapy (*n* = 6). g) The immunohistochemical staining of brain tumor sections on day 9 postimplantation using CRT, CD80, and CD8 antibodies. h) The i.n. combo treatment scheme. On day 4, 9, and 14 after tumor implantation, LCPN‐bearing mice were subject to 3 Gy radiotherapy and 6 h later i.n. injection with t‐NanoCpG (0.5 mg kg^−1^). i) Relative body weight (*n* = 7). j) Survival curves (*n* = 7). The plasma concentration of k) IFN‐*γ*, l) TNF‐*α*, and m) IL‐6 on day 15 in i.n. combo therapy (*n* = 7). *p < 0.05, **p < 0.01, ***p < 0.001, ****p < 0.0001.

The immunotherapeutic efficacy of t‐NanoCpG for glioma is expected to be further enhanced by combining chemo‐ or radio‐therapy that provides more tumor‐associated antigens (TAA). To prove this, we investigated the combination of t‐NanoCpG via i.v. or i.n. injection with radiotherapy for orthotopic LCPN mice. For i.v. combo treatment, on day 4, 6, and 8 after tumor implantation, LCPN‐bearing mice were first subject to 3 Gy radiotherapy and 6 h later i.v. injection of t‐NanoCpG (1 mg kg^−1^) (**Figure** **4**a). Remarkably, in contrast to monotherapy that showed considerable body weight loss within 30 days post‐tumor implantation, body weight changed little for the combo therapy (Figure 4b), suggesting that the combination of t‐NanoCpG (i.v.) and radiotherapy induces minimal adverse effects and high‐efficacy inhibition of glioma invasion. Interestingly, combo group revealed a markedly prolonged MST of 48 d, which was nearly doubled over PBS group and significantly longer than that for monotherapy groups (35 and 39 d for radiotherapy and t‐NanoCpG, respectively) (Figure 4c). The analyses of plasma proinflammatory cytokines revealed that combo group had significantly elevated IFN‐*γ* and TNF‐*α* compared with both monotherapy groups (Figure 4d,e). IL‐6 level was slightly higher than t‐NanoCpG (i.v.) group but significantly higher than radiotherapy (Figure 4f). The immunohistochemical staining of brain tumor sections on day 23 using CRT, CD80, and CD8 antibodies confirmed that the combo therapy induced evidently more ICD, activated APCs, and CD8^+^ T cells than the monotherapies (Figure 4g). It is noted that X‐ray radiation (3 Gy) at the present experimental settings did not compromise the BBB and improve brain delivery of t‐NanoCpG (Figure S12, Supporting Information). We further performed the combo therapy with i.n. injection of t‐NanoCpG and radiotherapy. On day 4, 9, and 14 after tumor implantation, LCPN‐bearing mice were subject to 3 Gy radiotherapy and 6 h later i.n. injection of t‐NanoCpG (0.5 mg kg^−1^) (Figure 4h). The results demonstrated again that combo therapy induced little adverse effects and significantly better therapeutic efficacy and survival benefits than the monotherapies (Figure 4i,j). The comparably higher plasma levels of IFN‐*γ*, TNF‐*α*, and IL‐6 observed for the combo group (Figure 4k–m) confirm that the combination of t‐NanoCpG and radiotherapy remarkably boosts the immunotherapy of malignant glioma. This study provides a robust and intelligent brain‐permeable CpG nano‐immunoadjuvant that potentiates the immunotherapy of glioma via minimally invasive administration.

The immunotherapy of malignant glioma has not met much success partly due to the lack of efficient and noninvasive brain delivery tools for immunoadjuvants to the tumors and cervical lymph nodes. CpG has demonstrated therapeutic potential against glioma via i.c., i.t., or local administration. These invasive approaches, however, generally demand application of immunosuppressant dexamethasone to avoid inflammation‐induced swelling and edema, which would substantially reduce T‐cell infiltration and response to tumor neoantigen vaccines. The clinical trials with i.c. or i.t. administration of free CpG in newly diagnosed or recurrent GBM patients failed to meet the endpoints.^[^ ^8^, ^28^ ^]^ The development of minimally invasive approaches is critical to the success of adjuvant‐based immunotherapy for malignant glioma. To the best of our knowledge, t‐NanoCpG represents the first CpG nano‐immunoadjuvant that is reported to trigger strong anti‐glioma therapy via noninvasive administration. t‐NanoCpG used either as a monotherapy or in a combo therapy with radiotherapy offers a new horizon for glioma treatment.

## 3 Conclusion

In summary, we have developed BBB‐permeable and glioma and CLN‐homing CpG nano‐immunoadjuvant (t‐NanoCpG) that enables intravenous and intranasal administration to strongly stimulate the maturation of dendritic cells and production of proinflammatory cytokines in vivo and to reprogram tumor immune microenvironment by enriching ICD, activated APCs and CD8^+^ T cells. t‐NanoCpG as a monotherapy induces significant survival benefits in orthotopic murine glioma LCPN‐bearing mice. The immunotherapy of glioma is further boosted by combining t‐NanoCpG with radiotherapy. Notably, the vehicles used in this study are based on poly(ethylene glycol), biodegradable polycarbonate and spermine, which are generally considered safe. The good safety, easy fabrication, minimal invasiveness, and high potency of t‐NanoCpG render it potentially appealing for clinical translation.

## Conflict of Interest

The authors declare no conflict of interest.

## Supporting information

Supporting Information

## Data Availability

The data that support the findings of this study are available from the corresponding author upon reasonable request.
