# Supplementary material for: Immunotherapy of Malignant Glioma by Noninvasive Administration of TLR9 Agonist CpG Nano‐Immunoadjuvant
Source: Adv Sci (Weinh). 2022 Mar 7;9(13):2103689. doi: 10.1002/advs.202103689 (PMC9069387; doi:10.1002/advs.202103689)
Supplement: Supplementary file 1 — Supporting Information [file ADVS-9-2103689-s001.pdf]

ADVANCED SCIENCE
Open Access

Supporting Information

for *Adv. Sci.*, DOI 10.1002/advs.202103689

Immunotherapy of Malignant Glioma by Noninvasive Administration of TLR9 Agonist CpG Nano-Immunoadjuvant

*Jingjing Wei, Di Wu, Songsong Zhao, Yu Shao, Yifeng Xia, Dawei Ni, Xinyun Qiu, Jinping Zhang, Jian Chen**, Fenghua Meng* and Zhiyuan Zhong**

Copyright WILEY-VCH Verlag GmbH & Co. KGaA, 69469 Weinheim, Germany, 2018.

Supporting Information

Immunotherapy of Malignant Glioma by Non-Invasive Administration of TLR9 Agonist CpG Nano-immunoadjuvant

Jingjing Wei, Di Wu, Songsong Zhao, Yu Shao, Yifeng Xia, Dawei Ni, Xinyun Qiu, Jinping Zhang, Jian Chen, Fenghua Meng*, and Zhiyuan Zhong**

J.J. Wei, S.S. Zhao, Y.F. Xia, Prof. F.H. Meng, D.W. Ni, X.Y. Qiu, Prof. Z.Y. Zhong
Biomedical Polymers Laboratory, College of Chemistry, Chemical Engineering and Materials Science, College of Pharmaceutical Sciences, and State Key Laboratory of Radiation Medicine and Protection, Soochow University, Suzhou 215123, P. R. China
E-mail: fhmeng@suda.edu.cn, zyzhong@suda.edu.cn

D. Wu, Prof. J. Chen
Institute of Functional Nano & Soft Materials (FUNSOM), Soochow University, Suzhou 215123, P. R. China
E-mail: chenjian@cibr.ac.cn

Y. Shao, Prof. J.P. Zhang
Institutes of Biology and Medical Sciences (IBMS), Soochow University, Suzhou 215123, P. R. China

Prof. J. Chen
Chinese Institute for Brain Research, Beijing; Research Unit of Medical Neurobiology, Chinese Academy of Medical Sciences (No. 2019RU003), Beijing, 102206, P. R. China

1. Materials

CpG ODN 1826 (CpG, 5'-TCCATGACGTTCCTGACGTT-3', Invivogen), Cy3-labeled CpG (CpG-Cy3, Genepharma), FITC-labeled CpG (CpG-FITC, Genepharma), Cy7-labeled CpG (CpG-Cy7, Sangon Biotech), ApoE peptide (LRKLRKRLLLRKLRKRLLC, 95%, GL Biochem) and dithiothreotol (DTT, 99%, Yeasen Biotech Co., Ltd.) were used without further purification. PEG-P(TMC-DTC)-sp (M_n = 5.0-(14.9-2.0)-0.2 kg/mol, spermine functionality: ~ 95%) and ApoE-PEG-P(DTC-TMC) (M_n = 7.5-(15.2-1.9) kg/mol, ApoE peptide functionality > 95%) were prepared according to our previous reports[1]. Other chemical reagents were purchased from Sinopharm Chemical Reagent Co., Ltd. and used as received.

Granulocyte-macrophage colony stimulating factor (GM-CSF, PeproTech, 315-03), macrophage colony stimulating factor (M-CSF, PeproTech, 315-02), and antibodies including anti-CD8-PE-Cy7 (Biolegend), anti-CD4-PerCP-Cy5.5 (eBioscience), anti-CD11c-FITC (Biolegend), anti-CD80-APC (eBioscience), anti-CD86-PE (eBioscience), rabbit mAb calreticulin (CRT, Abcam, ab92516), rabbit mAb CD8 (Abcam, ab217344), rabbit mAb CD80 (Abcam, ab254579) and Alexa Fluor® 633 goat anti-rabbit IgG (H&L) (Invitrogen, A21070) were used according to supplier's protocol. ELISA (Enzyme-linked immuno sorbent assay) kits including tumor necrosis factor alpha (TNF-α, Invitrogen, BMS607HS), interferon gamma (IFN-γ, Invitrogen, BMS609) and interleukin 6 (IL-6, CUSABIO, CSB-E04639m) as well as Micro-BCA protein assay kit (Pierce, Thermo Scientific, USA) were used as indicated by the manual.

2. Characterizations

^{1}H NMR spectra were measured on Unity Inova 400 spectrometer operating at 400 MHz using DMSO-d_6 as a solvent. Chemical shifts were calibrated in parts per million (ppm) referenced with respect to residual solvent (DMSO: δ 2.5). The size and size distribution, and zeta potential of polymersomes were determined by dynamic light scattering (DLS, Zetasizer Nano-ZS, Malvern Instruments) at 25 ºC equipped with a 633 nm He-Ne laser using back-scattering detection and with an electrophoresis U-shaped cell, respectively. NanoDrop One was used to determine the loading content of CpG in polymersomes. An FLS920 fluorescence spectrometer was applied for concentration determination of FITC-labeled CpG (CpG-FITC) and Cy3-labeled CpG (CpG-Cy3). ELISA assays were quantified by measuring the absorbance at a wavelength of 450 nm using a microplate reader (Thermo Scientific Varioskan LUX). Flow cytometry measurements were conducted on a flow cytometer (BD FACS Calibur, USA).

3. Preparation of CpG-loaded polymersomes (t-NanoCpG)

The ApoE peptide-functionalized CpG-loaded polymersomes (t-NanoCpG) were prepared by adding a DMF solution of ApoE-PEG-P(TMC-DTC) and PEG-P(TMC-DTC)-sp dropwise into HEPES buffer containing CpG, followed by dialysis. In a representative sample of 20 wt.% t-NanoCpG, DMF solution of ApoE-PEG-P(TMC-DTC) and PEG-P(TMC-DTC)-sp at a molar ratio of 20/80 (50 μL, total polymer concentration = 20 mg/mL) was injected into HEPES buffer (950 μL, 5 mM, pH 6.8) containing 110 μg of CpG under stirring for 10 min. The obtained dispersion was dialyzed (MWCO: 350 kDa) sequentially against HEPES for 2 h, HEPES/PB (1/1, v/v) for 1 h, and PB for 2 h with an exchange of fresh medium every hour. The size, size distribution and zeta potential of polymersomes and CpG concentrations were measured. The drug loading efficiency (DLE) and content (DLC) were calculated using the following equations:

$$\text{DLC (wt.\%)} = \frac{\text{weight of loaded CpG}}{\text{weight of polymers and loaded CpG}} \times 100$$

$$\text{DLE (\%)} = \frac{\text{weight of loaded CpG}}{\text{weight of CpG in feed}} \times 100$$

The non-targeted control NanoCpG was fabricated similarly to t-NanoCpG without adding ApoE-PEG-P(TMC-DTC). Nano and t-Nano were prepared similarly to NanoCpG and t-NanoCpG, respectively, without adding CpG.

The colloidal stability of t-NanoCpG at dilution with PB (10 mM, pH 7.4), incubation with 10% FBS, and storage at 4 °C was studied by tracking the changes in size and size distribution using Zetasizer Nano-ZS, and the CpG leakage during storage at 4 ºC for 7 days was measured using Nanodrop.

NanoCpG and t-NanoCpG loaded with FITC-labeled CpG (CpG-FITC) was used to study the in vitro release of CpG using dialysis method (Spectra/Pore, MWCO 12-14 kDa) at extracellular mimicking condition or acidic endo/lysosome mimicking conditions (n = 3). Typically, NanoCpG and t-NanoCpG (CpG concentration: 30 μg/mL) were added into dialysis tubes, and dialyzed against 20 mL PB (pH 7.4, 6.5 or 4.0). At desired time points, 5 mL dialysis medium was taken to determine the released SF using fluorophotometer based on a standard curve, and 5 mL fresh medium was replenished. The cumulative CpG release from nanoformulations were calculated as the ratio of cumulative released CpG in the dialysis tubes to the total amount.

4. In vitro study of BBB transcytosis of t-NanoCpG

BBB transcytosis capability of t-NanoCpG was evaluated with an in vitro BBB model

established by monolayer of murine bEnd.3 cells, which were cultured in DMEM medium (HyClone, USA) supplemented with 1 vol.% (100 IU/mL penicillin and 100 μg/mL streptomycin) and 10 vol.% fetal bovine serum (FBS, Gibco, USA) in a humidified atmosphere containing 5% CO_2 at 37 °C.

Briefly, 1.0×10^5 bEnd.3 cells were seeded on 24-well transwell membranes with a mean pore size of 1.0 μm and surface area of 0.33 cm^2 (Corning, USA) and the bottom of 24-well plates were immersed in DMEM medium for 24 h. The integrity of the bEnd.3 cell monolayer was characterized by measuring the trans-endothelial electrical resistance (TEER) with an epithelial voltmeter (Millicell-RES, Millipore, USA). Once the TEER was above 200 $\Omega \cdot cm^2$, CpG, NanoCpG and t-NanoCpG were added to the donor chamber using Cy3-labeled CpG (CpG-Cy3) for facile quantification (CpG-Cy3 concentration: 1 μg/mL) and co-cultured for 24 h (n = 3). At 6, 12 and 24 h, the bottom medium was collected and replenished with fresh medium. The fluorescence of collected bottom medium was measured to determine the amount of CpG. The transport ratios of CpG formulations from bEnd.3 cells were calculated as the ratio of CpG in the bottom medium to the total amount. The TEER of monolayer was kept above 200 $\Omega \cdot cm^2$ throughout the experiment to guarantee the integrity of the BBB model.

5. Activation of BMDCs and BMDM in vitro by flow cytometry

The bone marrow-derived dendritic cells (BMDCs) and bone marrow-derived macrophages (BMDM) were extracted from healthy C57BL/6 mice (6-week, female), and induced differentiation in vitro with GM-CSF and M-CSF, respectively. For activation evaluation, BMDCs or BMDM seeded in 6-well plate (5.0×10^5 cells/well) were incubated with Nano, t-Nano, CpG, NanoCpG or t-NanoCpG ([polymer]: 4 μg/mL, [CpG]: 0.4 μg/mL) at 37 °C (n = 3). After 24 h the culture medium was taken for determining the concentration of TNF-α and IL-6 using ELISA kits. The BMDCs were collected and stained with anti-CD11c-FITC, anti-CD80-APC and anti-CD86-PE antibodies, and BMDM were stained with anti-CD11b-FITC, anti-F4/80-PE, and anti-CD206-Alexa fluor® 647 antibodies. The fluorescence histograms were immediately recorded with a BD FACS flow cytometer and analyzed using FlowJo v10.

6. In vivo pharmacokinetics

Six-week-old female C57BL/6 mice were purchased from Beijing Vital River Laboratory Animal Technology Co., Ltd. and were housed in pathogen-free conditions at the Soochow University. All animal experiments were approved by the Animal Care and Use Committee of Soochow University (P. R. China) and all protocols of animal studies conformed to the Guide

for the Care and Use of Laboratory Animals.

In vivo pharmacokinetics of CpG formulations were studied with CpG-Cy3 for facile quantification. Free CpG, NanoCpG and t-NanoCpG (1 mg CpG equiv./kg) in 200 μL of PB were intravenously (i.v.) injected into healthy C57BL/6 mice (6-week, female) via tail veins (n = 3). At the set times, ca. 60 μL blood was taken from the retro-orbital sinus into heparinized tubes before centrifugation at 3000 rpm for 10 min. 20 μL plasma was taken immediately and incubated with 600 μL DMSO solution (containing 20 mM DTT) at 37 °C for 24 h to extract CpG-Cy3. CpG-Cy3 solutions of known concentrations underwent the same treatments for obtaining calibration curve. The concentrations of CpG-Cy3 in plasma were determined by fluorometry and plotted versus time. The elimination half-lives and area under the curve (AUC) were analyzed using software PK Solver.

7. Establishment of orthotopic murine LCPN glioma model

LCPN murine glioma cell line and luciferase-transfected LCPN cells (LCPN-Luc) were established by Prof. Dr. Jian Chen. Briefly, the LCPN cell was derived from a P5 male mouse neural stem cell with deletions of two most frequently mutated genes in human gliomas, P53 and NF1, by CRISPR/Cas9 technology.

Orthotopic murine LCPN glioma model was established in six-week-old female C57BL/6 mice by stereotactically injecting 5.0×10^4 LCPN cells in 5 μL PBS containing 25% matrigel into the right striatum using a 26-gauge Hamilton syringe (coordinates: 0.5 mm anterior, 1.9 mm lateral, and 3.1 mm deep) and the syringe remained for 5 min. Immunohistochemical staining of the slices of LCPN-bearing brain with anti-GFAP (a representative marker of glial cells) and anti-Ki67 (a representative marker of proliferating cells) showed $GFAP^+$ and $Ki67^+$ feature, confirming the glioma nature of LCPN cells (below).

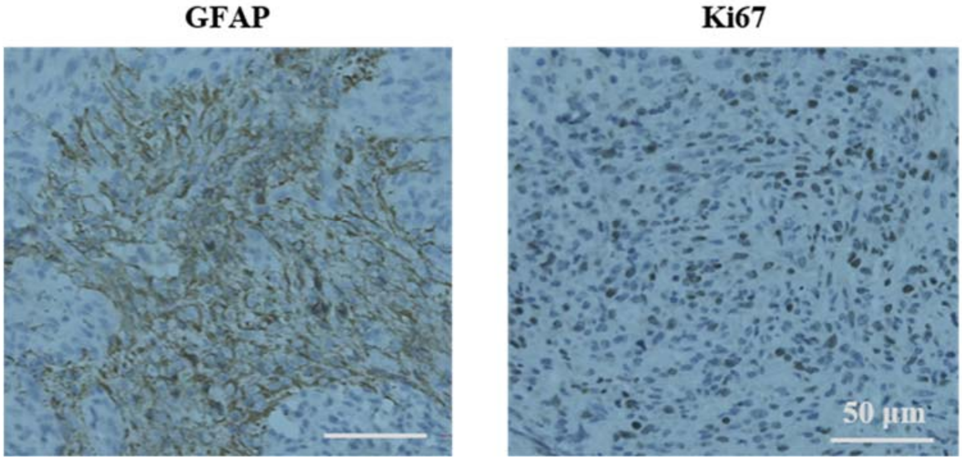

Orthotopic LCPN-Luc glioma model, established using the same method as above except 8.0×10^4 LCPN-Luc cells was injected, was applied to monitor the tumor progression and body weight change (n = 4). On day 4, 7, 10, 13, 19 and 23 post tumor-innoculation, luciferin potassium salt (75 mg/kg) was i.p. injected, and within 15 min the bioluminescence of animals was measured to monitor the tumor progression. The mice were weighed every two days. The bioluminescence intensity and body weight of the mice were relative to their values on D4.

8. In vivo biodistribution of CpG formulations

In vivo biodistribution of CpG formulations were studied with CpG-Cy3 in orthotopic murine LCPN glioma mice. Free CpG, NanoCpG and t-NanoCpG (1 mg CpG equiv./kg) in 200 µL PB were i.v. injected into LCPN-bearing mice via tail veins (n = 3). At 12 h post injection, main organs (heart, liver, spleen, lung and kidney), brain, glioma and cervical lymph nodes were dissected, weighed and homogenized in DMSO solution (containing 20 mM DTT) to extract CpG-Cy3. CpG-Cy3 solutions of known concentrations underwent the same treatment to draw calibration curves. The concentrations of CpG-Cy3 were determined by fluorometry and presented as percentage injected dose per gram of tissue (%ID/g).

9. The anti-glioma efficacy of CpG formulations by intravenous (i.v.) injection

The LCPN-bearing mice were randomly divided into six groups (n = 11): PBS, free CpG (1 mg/kg), NanoCpG (1 mg/kg) and t-NanoCpG (0.5, 1 and 2 mg/kg) and t-Nano (polymer: 10 mg/kg). On day 4, 6, 8 post-implantation, 200 µL CpG formulations were i.v. administered. At 24 h after the last injection, ca. 120 µL blood was taken from the orbit into heparinized tubes and centrifugated immediately to get plasma, the plasma concentrations of IFN-γ, TNF-α and IL-6 were measured using ELISA kits according to manufacturer's manuals (n = 4). On day 9 post-implantation, four mice from the group of PBS, CpG, NanoCpG or t-NanoCpG (1 mg/kg) were sacrificed to extract tumors and lymph nodes for immune cell analyses and tumor immunohistochemical analyses (n = 1), and blood for blood biochemistry and blood routine analysis (n = 3), respectively. On day 23, the mice in PBS group displayed signs of neurological deficits, then one mouse from each group was sacrificed and glioma-bearing brain was excised for hematoxylin and eosin (H&E) staining (n = 1). The rest mice were monitored for body weight and survival curves (n = 6). The body weight of the mice was relative to their values on D4.

To analysis the immune environment of tumors and lymph nodes, on day 9, the tumors were sliced and the antigen-releasing tumor cells were stained by rabbit anti-mouse CRT

antibody (Abcam, ab2907). The tumor infiltrating $CD8^+$ T cells and APCs were stained by rabbit mAb CD8 antibody (Abcam, ab217344) and CD80 antibody (Abcam, ab254579), respectively. The secondary antibodies used were goat anti-rabbit IgG H&L (Alexa Fluor® 647) for optical microscopy observation.

The extracted tumors and lymph nodes were cut into small pieces and homogenized in cold PBS on ice bath to obtain single-cell suspensions. After the red blood cells were lysed, the immune cells in the single-cell suspensions were labeled with CD11c, CD80, CD86, CD8 and CD4 antibodies according to manufacturer's protocols, and measured with FACS flow cytometer (BD Bioscience) and analyzed using FlowJo version 10.

10. The anti-glioma efficacy of CpG formulations by intranasal (i.n.) injection

The LCPN-bearing mice were randomly divided into four groups (n = 7): PBS, free CpG, NanoCpG and t-NanoCpG at CpG dosage of 0.5 mg/kg. On day 4, 9, 14 post-implantation, 8 μL CpG formulations were i.n. administered. At 24 h after the last i.n. injection, the plasma concentration of IFN-γ, TNF-α and IL-6 were determined as described above (n = 7). The mice were monitored for body weight and survival curves. The body weight of the mice was relative to their values on D4.

11. The anti-glioma efficacy of the combination of i.v. t-NanoCpG and radiotherapy

The LCPN-bearing mice were randomly divided into four groups (n = 7): PBS, t-NanoCpG (i.v.), X-ray (3 Gy) and the combo of t-NanoCpG (i.v). and X-ray at CpG dosage of 1 mg/kg. On day 4, 6 and 8 post-implantation, the mice were irradiated with 3 Gy X-ray, and 6 h later t-NanoCpG was i.v. administered. At 24 h after the last i.v. injection, the plasma concentration of cytokines were determined (n = 6). On day 9, the tumor slices were stained with anti-CRT, anti-CD8 or anti-CD80 followed by goat anti-rabbit IgG H&L (Alexa Fluor® 647) as secondary antibody. The rest mice were monitored for body weight and survival curves (n = 6). The body weight of the mice was relative to their values on D4.

To investigate the effect of X ray on the brain delivery, on day 10 post-implantation t-NanoCpG loaded with Cy7 labeled CpG (1 mg CpG/kg, 0.5 μg Cy7 per mouse) was i.v. injected into orthotopic LCPN-bearing mice that received nothing or 3 Gy X-ray irradiation 6 h ago. In vivo fluorescence image of the mice were scanned using an IVIS II system (n = 3).

12. The anti-glioma efficacy of the combination of i.n. t-NanoCpG and radiotherapy

The LCPN-bearing mice were randomly divided into four groups (n = 7): PBS, t-NanoCpG (i.n.), X-ray (3 Gy) and the combo of t-NanoCpG (i.n.) and X-ray at CpG dosage of 0.5 mg/kg. On day 4, 9 and 14, LCPN tumor-bearing mice were irradiated with 3 Gy X-ray, and 6 h later

t-NanoCpG was i.n. administered. At 24 h after final i.n. injection, the plasma cytokines were determined (n = 7). The mice were monitored for body weight and survival curves. The body weight of the mice was relative to their values on D4.

13. The anti-GL261 efficacy of CpG formulations by intravenous (i.v.) injection

Orthotopic murine GL261 glioma model was established in six-week-old female C57BL/6 mice by stereotactically injecting GL261-Luc cells (5.0×10^4) in 5 μL of PBS containing 25% matrigel into the right striatum using a 26-gauge Hamilton syringe (coordinates: 0.5 mm anterior, 1.9 mm lateral, and 3.1 mm deep) and the syringe remained for 5 min.

To study the therapeutic activity of CpG formulations on GL261-Luc-bearing mice, the mice were divided into five groups (n = 6) on day 4 post tumor implantation: PBS, free CpG, NanoCpG, t-NanoCpG (CpG: 1 mg/kg) and t-Nano (polymer: 10 mg/kg). 200 μL of CpG formulations were i.v. administered on day 4, 6, and 8 post-implantation. On day 4, 7 and 10, luciferin potassium salt (75 mg/kg) was i.p. injected, and within 15 min the bioluminescence of animals was measured to monitor the tumor progression. The bioluminescence intensity and body weight of the mice were relative to their values on D4. On day 23, all alive mice from PBS (n = 6), CpG (n = 4), NanoCpG (n = 6), t-NanoCpG (n = 6) or t-Nano (n = 3) groups were sacrificed to isolate tumors for immune cell analyses. The extracted GL261-Luc tumors were treated and measured as LCPN model. The immune cells in the single-cell suspensions were labeled with CD11c, CD3, CD8 and CD4 antibodies, and measured with FACS flow cytometer.

14. Statistical analysis

Data was represented as means ± standard deviation (SD). The significant differences among groups were determined using GraphPad Prism 8 by One-way ANOVA. For survival analysis, One-way ANOVA using the log-rank (Mantel-Cox) test was applied. For others, One-way ANOVA using the Tukey's multiple comparisons test was applied. * $p < 0.05$ meant significant, ** $p < 0.01$, *** $p < 0.001$ and **** $p < 0.0001$ meant highly significant.

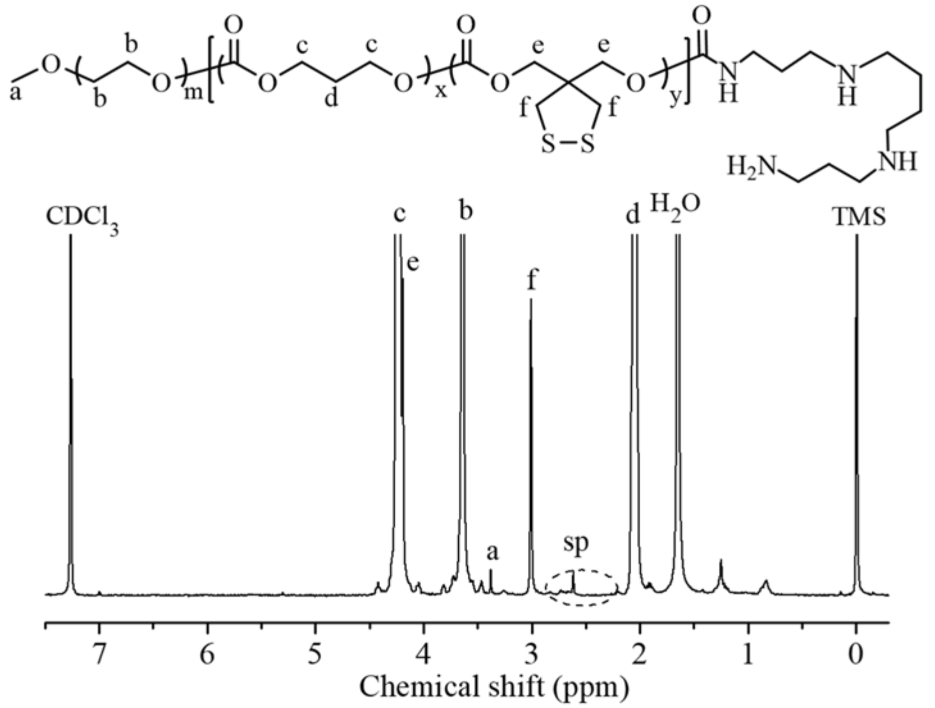

Figure S1. ^{1}H NMR spectrum (400 MHz, $CDCl_3$) of PEG-P(TMC-DTC)-sp.

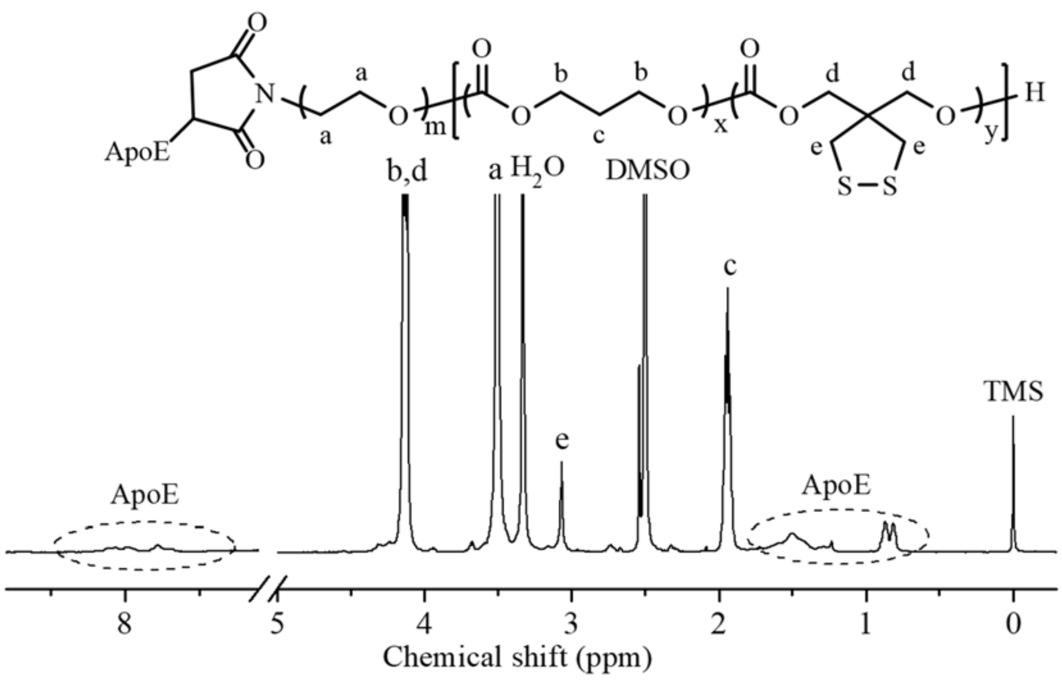

Figure S2. ^{1}H NMR spectrum (400 MHz, DMSO-d_6) of ApoE-PEG-P(TMC-DTC).

Table S1. Characterization of PS-CpG and ApoE-PS-CpG.

Polymersomes	DLC (wt.%)		DLE[a] (%)	Size[b] (nm)	PDI[b]	Zeta potential[b] (mV)
	Theory	Determined[a]				
t-NanoCpG	10	10.0	100.0	44	0.16	-5.2
	15	14.0	92.2	46	0.18	-5.1
	20	18.9	93.2	45	0.19	-7.8
NanoCpG	10	10.0	100.0	50	0.12	-3.4
	15	14.2	93.8	49	0.11	-4.6
	20	19.2	95.0	51	0.06	-7.2

[a] measured by nanodrop; [b] measured by DLS at 25 °C in PB (pH 7.4, 10 mM).

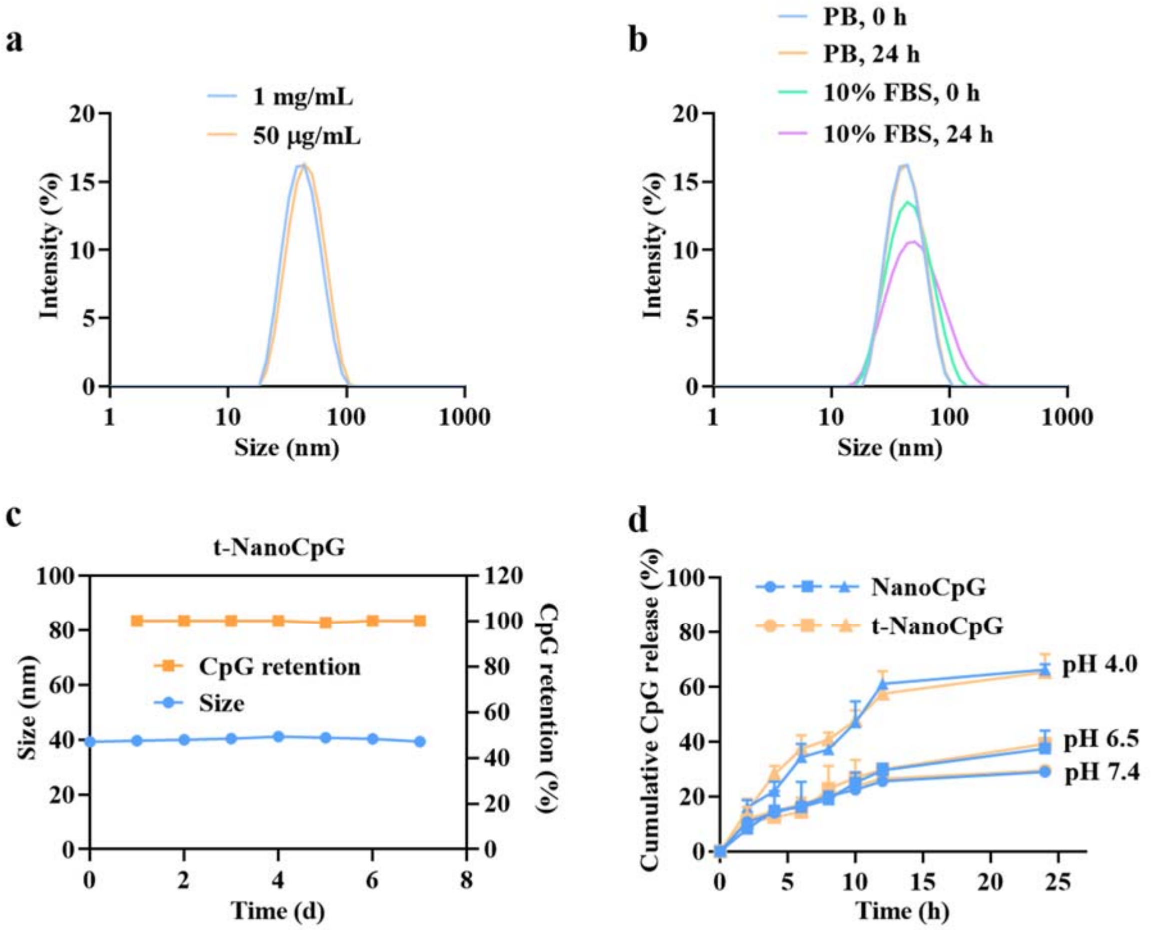

Figure S3. The stability study of t-NanoCpG against dilution (a), 10% FBS (b) and storage over 7 days at 4 °C (c). d) *In vitro* drug release study of NanoCpG and t-NanoCpG with FITC-labeled CpG at pH 7.4, 6.5 or 4.0 (n = 3).

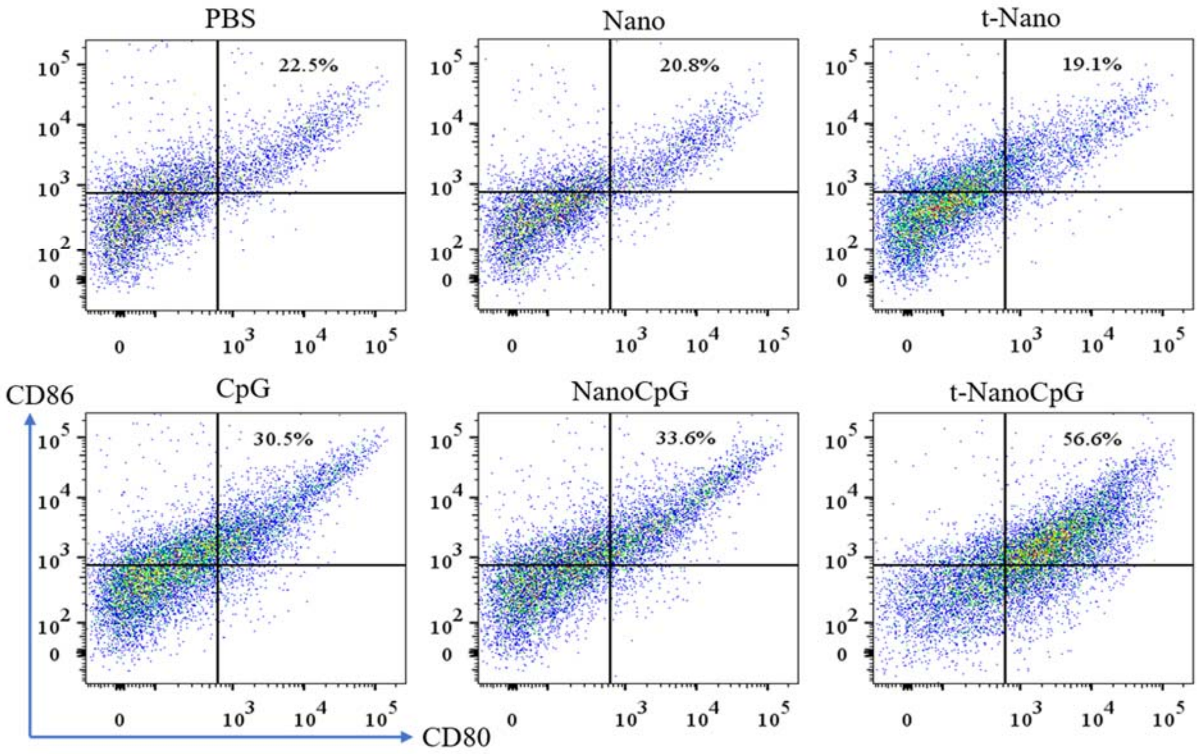

Figure S4. Representative flow cytometric graphs of BMDC maturation ($CD80^+CD86^+$) (gated on $CD11c^+$) in vitro stimulated by PBS, Nano, t-Nano, CpG, NanoCpG or t-NanoCpG (n = 3). Polymer concentration: 4 µg/mL. CpG concentration: 0.4 µg/mL.

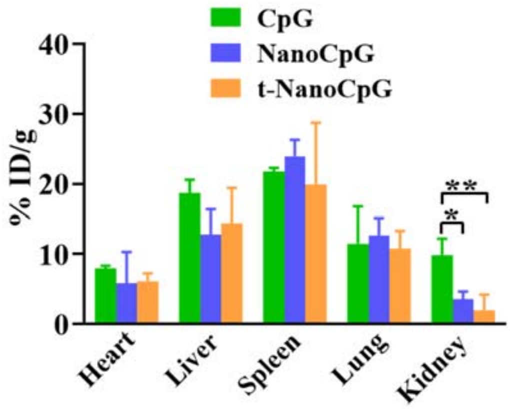

Figure S5. The in vivo bio-distribution of CpG in main organs (heart, liver, spleen, lung and kidney) of LCPN-bearing mice at 8 h post intravenous injection of Cy3-labeled free CpG, NanoCpG or t-NanoCpG (n = 3).

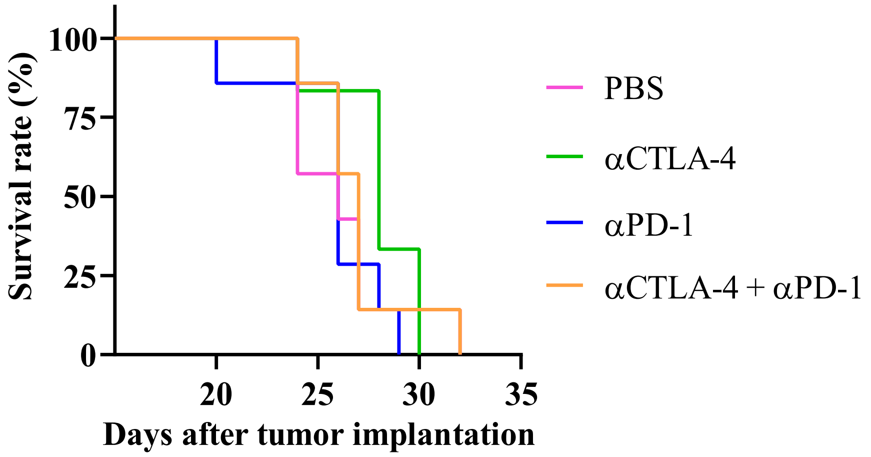

Figure S6. Intraperitoneal injection of anti-CTLA-4 and/or anti-PD-1 has no survival benefit in orthotopic murine LCPN glioma model. LCPN-bearing mice were intraperitoneally injected with PBS (n = 7), anti-CTLA-4 (8 mg/kg on day 10, 4 mg/kg on day 13, 16, and 19, n = 6), anti-PD-1 (6 mg/kg, n = 7) or anti-CTLA-4 + anti-PD-1 (n = 7) on day 10, 13, 16, and 19 after tumor implantation.

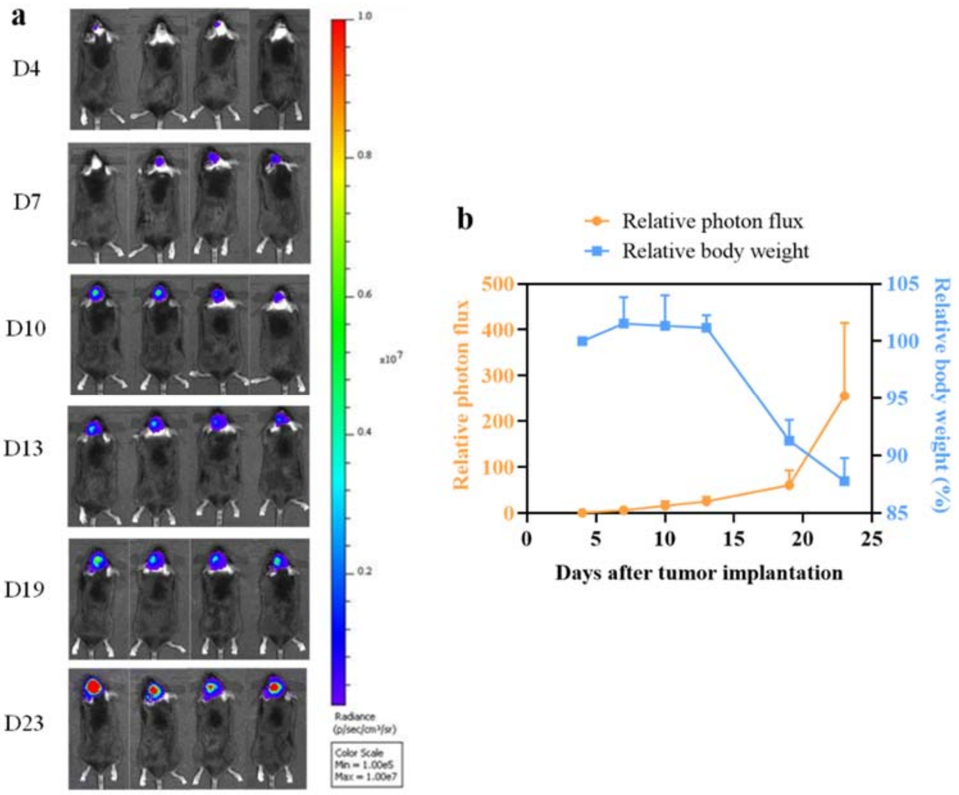

Figure S7. *In vivo* bioluminescence study on day 4, 7, 10, 13, 19 and 23 post tumor implantation of the mice bearing orthotopic LCPN-Luc glioma (n = 4). (a) *In vivo* bioluminescence images and (b) relative tumor bioluminescence intensity and relative body weight in time.

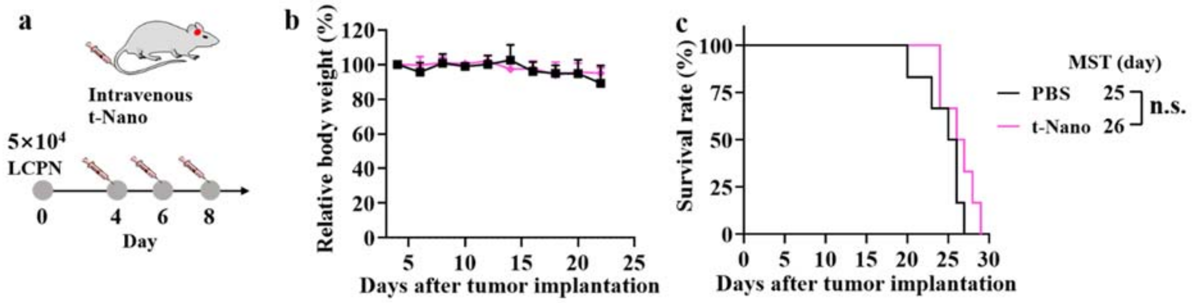

Figure S8. Immunotherapeutic efficacy of empty polymersomes t-Nano by intravenous injection in orthotopic LCPN model (n = 6). a) The administration scheme. LCPN-bearing mice were i.v. injected with t-Nano (polymer: 10 mg/kg) on day 4, 6, and 8 after tumor implantation. b) Relative body weight and c) survival curves of the mice.

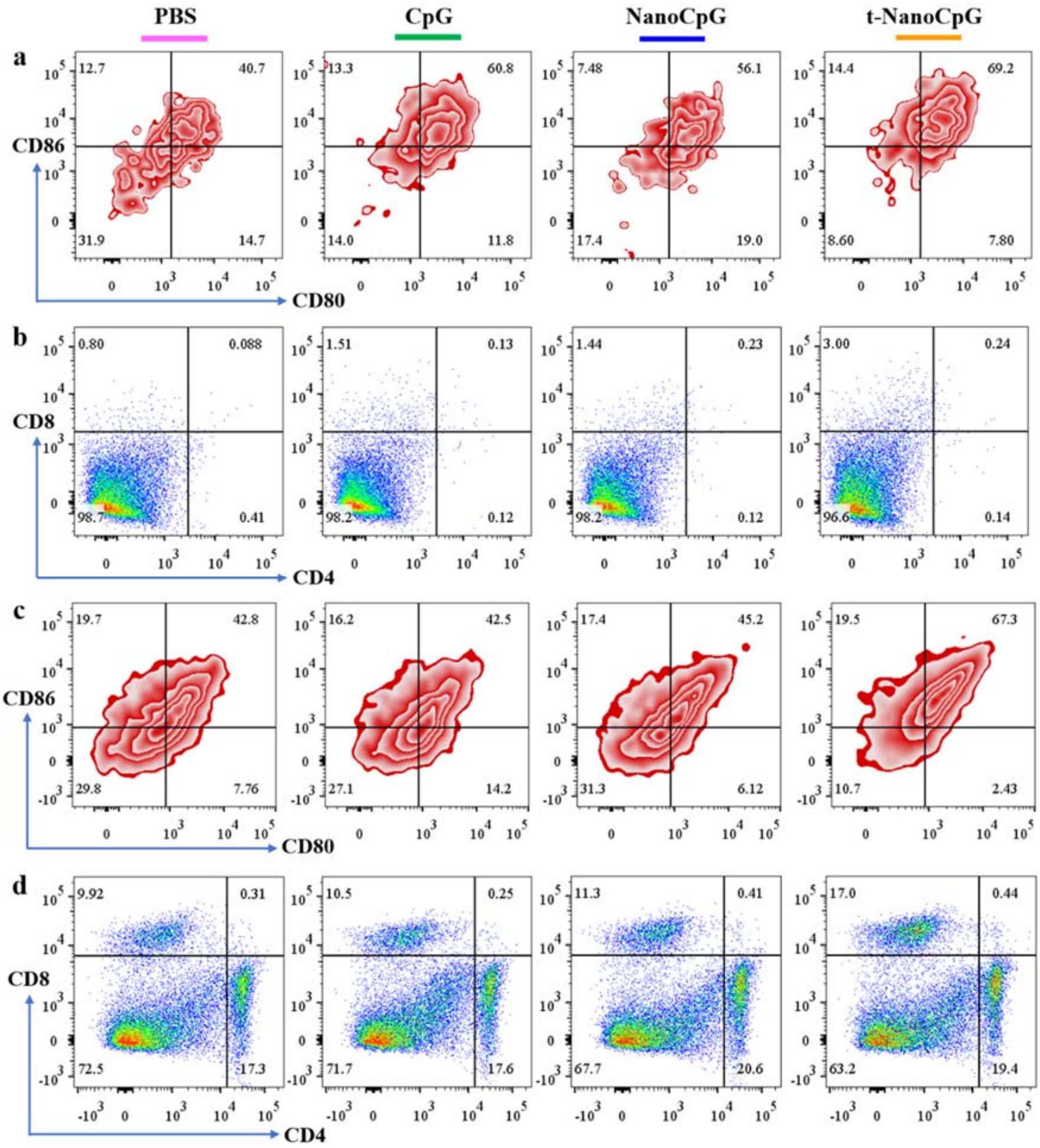

Figure S9. Immune environment analysis of tumors (a, b) and lymph nodes (c, d) of orthotopic LCPN-bearing mice after immunotherapy with CpG formulations by intravenous injection as in Figure 2a. The representative flow cytometric graphs of mature DCs (CD80$^+$CD86$^+$) (gated on CD11c$^+$) and CD8$^+$ T cells and CD4$^+$ T cells in tumors (a, b) and lymph nodes (c, d), respectively (n = 3). PBS, CpG, NanoCpG or t-NanoCpG was given on day 4, 6, 8 post-implantation at a dosage of 1 mg/kg.

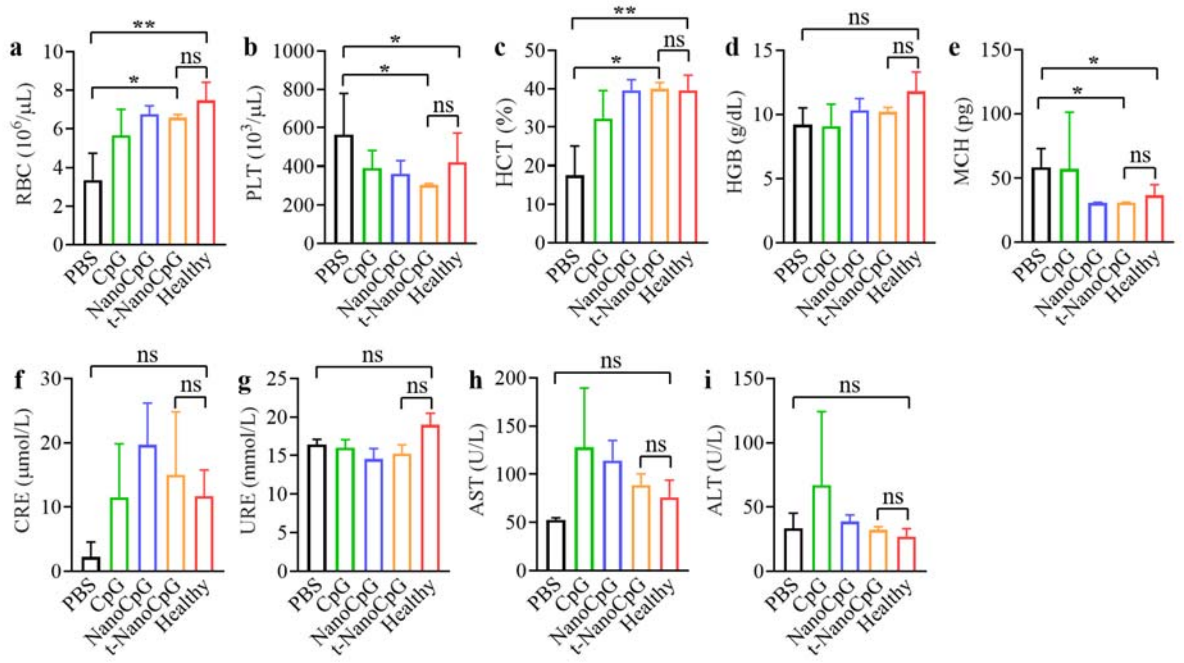

Figure S10. Blood biochemistry and blood routine analysis (n = 3) of the mice received t-NanoCpG, NanoCpG or free CpG treatment (CpG dosage: 1 mg/kg) via intravenous injection as showed in Figure 2a on day 9 after tumor implantation. a) Red blood cells (RBC). b) Platelets (PLT). c) Hematocrit (HCT). d) Hemoglobin (HGB). e) Mean corpuscular hemoglobin (MCH). f) Creatinine (CRE). g) Urea (URE). h) Aspartate transaminase (AST). i) Alanine transaminase (ALT).

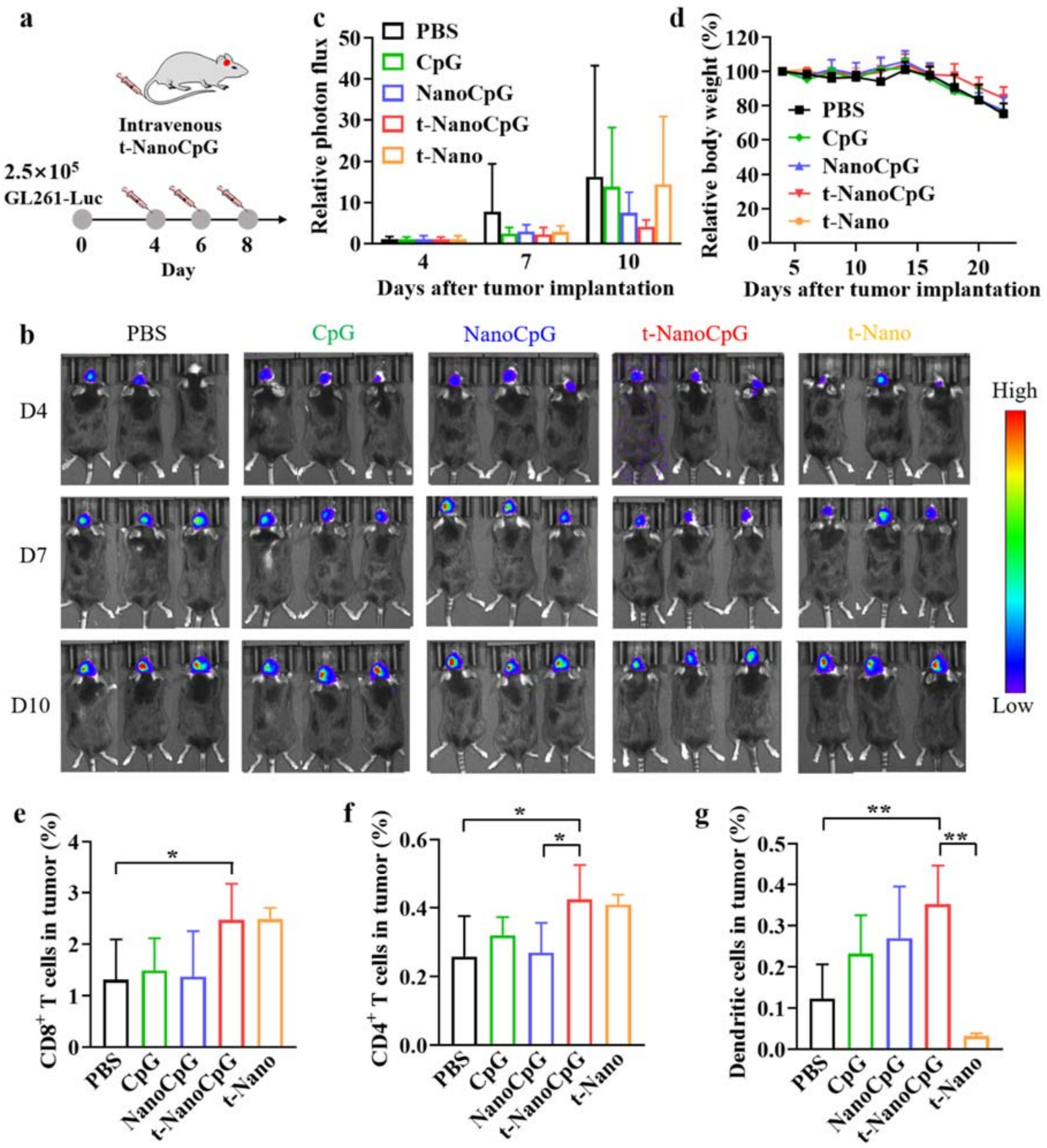

Figure S11. Intravenous injection of t-NanoCpG induces enhanced immunotherapeutic efficacy in orthotopic GL261 model. a) Intravenous administration scheme. GL261-bearing mice were i.v. injected with CpG, NanoCpG, t-NanoCpG (CpG dosage: 1 mg/kg) or t-Nano (polymer dosage: 10 mg/kg) on day 4, 6, and 8 after tumor implantation. b) The *in vivo* bioluminescence images of three representative mice of each group on day 4, 7 and 10 post tumor implantation (n = 6). c) The semi-quantitative analysis of tumor bioluminescence relative to their initial values on day 4 (n = 6). d) Relative body weight (n = 6). e-g) Analysis of immune cells in tumors of orthotopic GL261-bearing mice treated with PBS (n = 6), CpG (n = 4), NanoCpG (n = 6), t-NanoCpG (n = 6) or t-Nano (n = 3). Percentages of $CD8^+$ T cells (e), $CD4^+$ T cells (f) and DCs ($CD11c^+$) (g) in tumors on day 23 after tumor implantation.

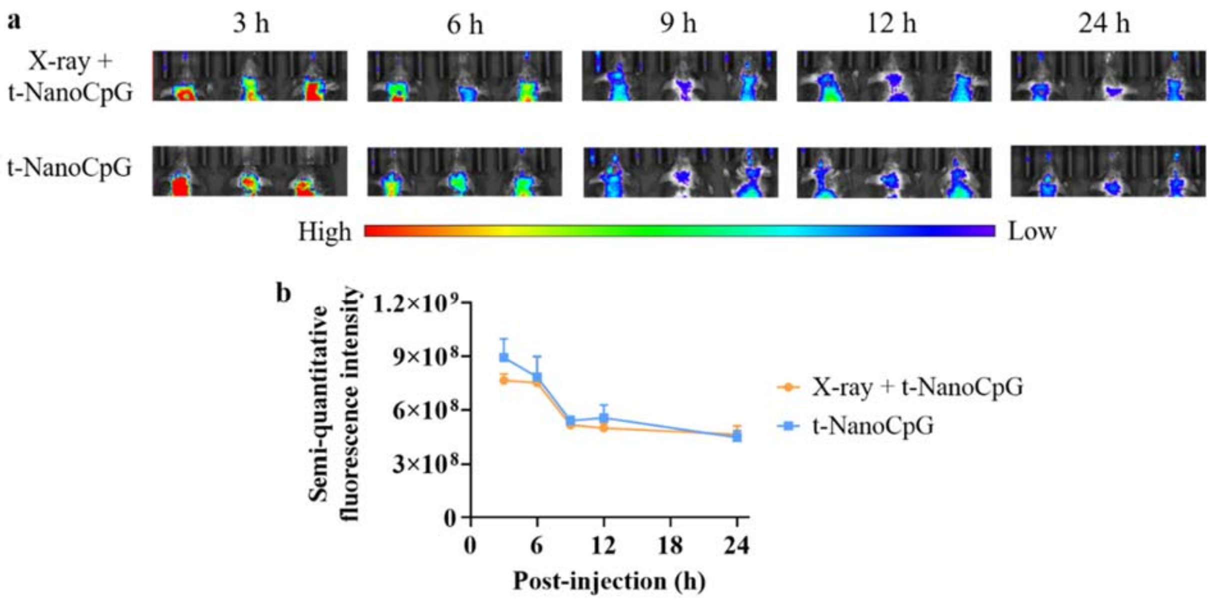

Figure S12. The brain *in vivo* NIR imaging a) and relative brain fluorescence intensity b) of orthotopic LCPN-bearing mice treated with intravenous injection of t-NanoCpG loaded with Cy7-labeled CpG at 6 h after 3 Gy X ray irradiation (n = 3).

References

[1] a) H. Qin, Y. Jiang, J. Zhang, C. Deng, Z. Zhong, *Mol. Pharm.* **2019**, *16*, 3711; b) Y. N. Zhong, F. H. Meng, W. Zhang, B. Li, J. C. M. van Hest, Z. Y. Zhong, *J. Control. Release* **2020**, *320*, 421.
